# *Streptococcus suis* infective endocarditis in patients with *Streptococcus suis* bacteremia: a retrospective study of prevalence and outcomes

**DOI:** 10.1186/s12879-024-10180-y

**Published:** 2024-11-14

**Authors:** Pongsira Kedsawadevong, Sirichai Jamnongprasatporn, Nithima Ratanasit

**Affiliations:** 1 Department of Medicine, Faculty of Medicine Siriraj Hospital, Mahidol University, Bangkok, Thailand; 2 Division of Cardiology, Department of Medicine, Faculty of Medicine Siriraj Hospital, Mahidol University, Bangkok, Thailand

**Keywords:** *Streptococcus suis* infective endocarditis, *Streptococcus suis* bacteremia, Prevalence, Outcomes

## Abstract

**Background:**

*Streptococcus suis (S. suis)* is a zoonotic disease that is transmitted to humans via contact or oral route. Although the major clinical presentation of this pathogen is known to be meningitis, *S. suis* infective endocarditis (IE) has recently emerged as a clinical manifestation of increasing interest. Echocardiography may be an underutilized modality for evaluating patients with *S. suis* bacteremia.

**Objective:**

The primary objective was to estimate the prevalence of *S. suis* IE in patients with *S. suis* bacteremia. The secondary objective was to investigate the predictors, echocardiographic features, and clinical outcomes of *S. suis* IE in patients with *S. suis* bacteremia.

**Materials and methods:**

This single-center retrospective study was conducted at Siriraj Hospital – Thailand’s largest university-based tertiary referral center. Adult patients (aged > 18 years) who were admitted to our center with confirmed diagnosis of *S. suis* bacteremia during January 2007 to September 2023 were included. Prevalence is reported as percentage and confidence interval. Baseline characteristics and clinical manifestation were compared between the IE and non-IE groups. Factors found to be statistically significant were further analyzed using binary logistic regression analysis to identify univariate predictors of *S. suis* IE.

**Result:**

A total of 71 patients with *S. suis* bacteremia were included in this study. The prevalence of *S. suis* IE was 26.8% (95% confidence interval: 17.85–38.05). Perivalvular complications and significant valvular regurgitation were found in 52.6% and 80.0% of patients, respectively. Thirteen of 19 patients (68.4%) required valvular surgery according to standard guidelines. By univariate analysis, dyspnea, new murmur, immunologic phenomenon, and heart failure were predictors of *S. suis* IE in patients with *S. suis* bacteremia.

**Conclusion:**

The results of this study revealed a sizable prevalence of *S. suis* IE in patients with *S. suis* bacteremia, and there were high rates of both valvular damage and perivalvular complications. Our results strongly suggest that echocardiography may be indicated to evaluate for *S. suis* IE in patients diagnosed with *S. suis* bacteremia. Reclassification of *S. suis* from an atypical organism to a typical organism should be considered.

## Introduction

*Streptococcus suis (S. suis)* is a Gram-positive alpha-hemolytic diplococci bacterium that is most commonly found in the respiratory tract of pigs. The most common mode of transmission to humans is via contact or oral route. This pathogen is increasingly recognized as a cause of serious infection globally, and especially in Southeast Asia. Many clinical syndromes have been described, including bacteremia, meningitis, septic arthritis, and infective endocarditis (IE) [1–5]. Previous studies reported that *S. suis* IE develops in 6.4–25.6% of *S. suis* infection cases [1, 2, 6]. The aggressive nature of this bacterium can result in a significant rate of valvular destruction, perivalvular complications, and distal embolization [4, 7]. Additionally, a substantial proportion of patients were reported to require valvular surgery as a result of these complications [4, 7]. Despite the sizable prevalence and poor clinical outcomes of *S. suis* IE, there are still no recommendations regarding routine echocardiography in cases of *S. suis* bacteremia. This is in contrast to the recommendations in the 2023 European Society of Cardiology (ESC) guideline in cases of *Staphylococcus aureus*, *Enterococcus faecalis*, and some *Streptococcus* spp. bacteremia [8]. 

The primary aim of this study was to estimate the prevalence of *S. suis* IE in patients with *S. suis* bacteremia. The secondary aim was to investigate the predictors, echocardiographic features, and clinical outcomes of *S. suis* IE in patients with *S. suis* bacteremia.

## Materials and methods

### Study design and participants

This single-center retrospective study enrolled patients with confirmed *S. suis* bacteremia who were admitted to the Internal Medicine Ward of Siriraj Hospital (Bangkok, Thailand) during 1 January 2007 to 30 September 2023. The Faculty of Medicine Siriraj Hospital, Mahidol University is Thailand’s largest university-based national tertiary referral center.

Patients aged older than 18 years who were admitted to Siriraj Hospital with at least one blood culture showing positive for *S. suis* were eligible for inclusion. IE included both ‘definite IE’, defined as two major or one major plus three minor criteria, and ‘possible IE’, defined as one major and one minor criteria, as described in the 2015 ESC guideline [9]. Transthoracic echocardiography (TTE) and transesophageal echocardiography (TEE) were performed according to the discretion of the primary physician. Pregnant women were excluded since these patients were admitted to the obstetrics ward, and an obstetrician was their primary doctor. The protocol for this study was approved by the Siriraj Institutional Review Board (SIRB) of the Faculty of Medicine Siriraj Hospital, Mahidol University, Bangkok, Thailand. (COA no. Si935/2023). Written informed consent was not obtained from study subjects due to the retrospective and anonymity-preserving design of our study.

### Measurement and outcomes

Patient data were searched using the keyword ‘*Streptococcus suis*’ from our center’s laboratory blood culture database. There were 71 patients with *S. suis* bacteremia included in the study. Baseline characteristics, echocardiographic results, and clinical outcomes were reviewed from patient electronic medical records. The primary outcome was the prevalence of *S. suis* IE in patients with *S. suis* bacteremia. The secondary outcomes were echocardiographic features, predictors, and outcomes of *S. suis* IE.

### Statistical analysis

All statistical analyses were performed using SPSS Statistics for Windows v. 26 (SPSS Inc., Chicago, IL, USA). Descriptive statistics were used to summarize patient demographic and baseline characteristics. Normally distributed continuous variables are described as mean ± standard deviation (SD), whereas non-normally distributed continuous variables are reported as median and interquartile range [IQR]. Student’s *t*-test was used to compare normally distributed continuous data, and Mann-Whitney U test was used for comparisons of non-normally distributed continuous data. Categorical variables are presented as number of cases and percentage. Chi-square test or Fischer’s exact test was used (according to the size of the sample being compared) to compare categorical data. Comparisons with a *p*-value lower than 0.05 were considered to be statistically significant. Factors found to be statistically significant in bivariate analysis were further analyzed using binary logistic regression analysis to identify univariate predictors of *S. suis* IE in patients with *S. suis* bacteremia. The results of univariate logistic regression analysis are expressed as odds ratio (OR) and 95% confidence interval (95%CI). Any predictors for which a crude OR could not be calculated were estimated using the analysis of binary data method (David R. Cox and E. J. Snell method) with + 0.5 smoothing to calculate the empirical logit transform to estimate the OR.

## Results

**Fig. 1:**
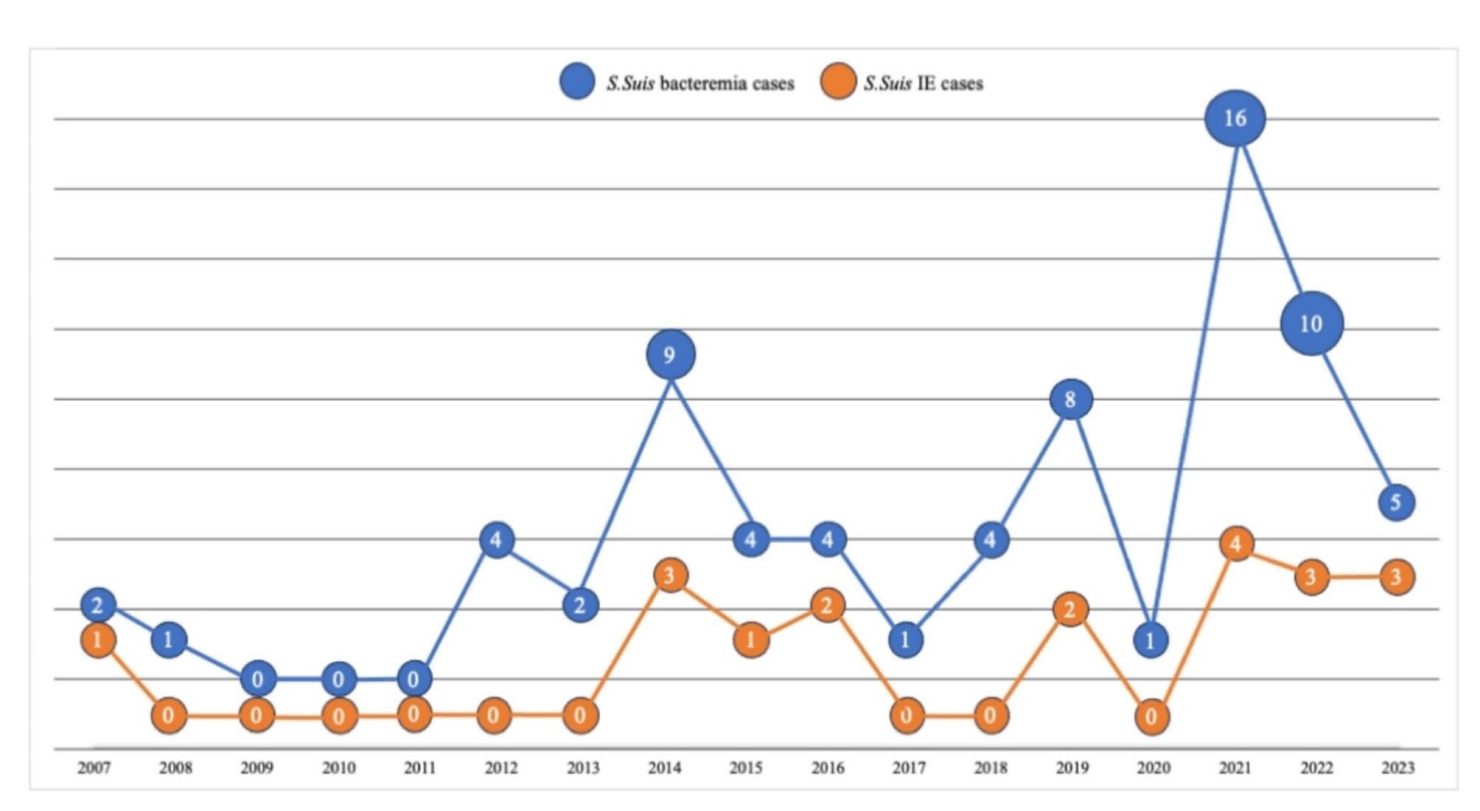
Number of patients with *S. suis* IE and *S. suis* bacteremia admitted to Siriraj Hospital from January 1st, 2007 to September 30th, 2023

A total of 71 patients were enrolled in the study. The mean age of patients was 59.9 ± 15.1 years, and 56.3% were male. Nineteen of 71 *S. suis* bacteremia patients (26.8%, 95%CI: 17.85–38.05) had *S. suis* IE. Patient baseline characteristics, presenting symptoms, and clinical syndromes are shown in Table 1. Approximately half of patients had hypertension and approximately one-fifth had diabetes. Fever was the most common clinical manifestation in the total study sample (85.8%), whereas dyspnea, immunologic phenomena, new murmurs, and heart failure were more prevalent in the IE group. IE was the second most common clinical syndrome in our *S. suis* bacteremia cohort. There was an observed trend towards an increased incidence of *S. suis* bacteremia over time that corresponded to an increased incidence of with *S. suis* IE, and the peak incidence was observed in 2021 (Fig. 1).

**Table 1:** Baseline characteristics, presenting symptoms, and clinical syndromes

	All patients (*n* = 71)	IE (*n* = 19)	No IE (*n* = 52)	*p*-value
**Baseline characteristics**				
- Age (years)	59.9 ± 15.1	55.5 ± 15.5	61.5 ± 14.8	0.140
- Sex: male	40 (56.3%)	10 (52.6%)	30 (57.7%)	0.703
- Height (cm)	161.2 ± 9.8	162.8 ± 8.0	160.6 ± 10.5	0.407
- Weight (kg)	60.3 ± 10.3	60.1 ± 9.9	60.3 ± 10.7	0.922
- BMI (kg/m²)	23.1 ± 3.5	22.6 ± 3.0	23.4 ± 3.7	0.416
- Hypertension	34 (47.9%)	6 (31.6%)	28 (53.8%)	0.096
- Diabetes mellitus	13 (18.3%)	2 (10.5%)	11 (21.2%)	0.491
**Presenting symptoms**				
- Fever	61 (85.9%)	18 (94.7%)	43 (82.7%)	0.270
- Dyspnea	16 (22.5%)	9 (47.4%)	7 (13.5%)	***0.008***
- Seizure	3 (4.2%)	0 (0.0%)	3 (5.8%)	0.559
- Headache	17 (24.3%)	2 (11.1%)	15 (28.8%)	0.203
- New murmur	15 (21.1%)	15 (78.9%)	0 (0.0%)	***< 0.001***
- Immunologic phenomenon	3 (4.2%)	3 (15.8%)	0 (0.0%)	***0.017***
- Vascular phenomena	2 (2.8%)	2 (10.5%)	0 (0.0%)	0.069
- Septic shock	8 (11.3%)	3 (15.8%)	5 (9.6%)	0.434
- Stroke/septic emboli	5 (7.0%)	2 (10.5%)	3 (5.8%)	0.605
- Heart failure	10 (14.1%)	9 (47.4%)	1 (1.9%)	***< 0.001***
**Organ-specific infection**				
- Meningitis	22 (31.0%)	4 (21.1%)	18 (34.6%)	0.329
- Intra-abdominal infection	4 (5.6%)	1 (5.3%)	3 (5.8%)	1.000
- Skin and soft tissue	3 (4.2%)	0 (0.0%)	3 (5.8%)	0.559
- Osteomyelitis	7 (9.9%)	3 (15.8%)	4 (7.7%)	0.375
- Arthritis	11 (15.5%)	2 (10.5%)	9 (17.3%)	0.715
- Pneumonia	4 (5.6%)	2 (10.5%)	2 (3.8%)	0.289
- Primary bacteremia	18 (25.3%)	0 (0.0%)	18 (34.6%)	***0.002***

Data are expressed as mean ± standard deviation or number and percentage

A *p*-value < 0.05 indicates statistical significance

Abbreviations: BMI: body mass index; IE: infective endocarditis

Univariate regression analysis revealed four factors to be significantly associated with *S. suis* IE, including dyspnea, new murmur, immunologic phenomenon, and heart failure (all *p* < 0.05). It should be noted that since new murmurs and immunologic phenomena were not found in the non-IE group, crude OR values could not be calculated for these factors. As a result, an analysis of binary data using + 0.5 smoothing to calculate the empirical logit transform was applied to estimate the crude OR for these two factors (Table 2). Multivariate regression analysis of the factors found to be significant in univariate regression analysis could not be performed due to the small sample size and possible unfavorable interaction between or among factors. As a result, we were able to identify significant predictors of *S. suis* IE, but not independent predictors of *S. suis* IE.

**Table 2:** Univariate analysis for factors that significantly predict IE in *S. suis* bacteremia

Factors	Univariate analysis		
	Crude OR	95%CI	*p*-value
Dyspnea	5.78	1.74–19.25	***0.008***
New murmur	364.67*	18.45-7093.59	***< 0.001***
Immunologic phenomenon	22.27*	1.09-453.86	***0.017***
Heart failure	45.90	5.22-403.75	***< 0.001***

A *p*-value < 0.05 indicates statistical significance

*Analysis of binary data using + 0.5 smoothing to calculate the empirical logit transform (David R. Cox and E. J. Snell) was applied to estimate the odds ratio

Abbreviations: 95%CI: 95% confidence interval; IE: infective endocarditis; OR: odds ratio; *S. suis*: *Streptococcus suis*

TTE and TEE was performed in 30 (42.3%) and 7 (9.9%) of 71 patients, respectively. In the IE group, TTE and TEE was performed in 19 (100%) and 7 (36.8%) patients, respectively. In the non-IE group, TTE was performed in 11 (21.2%) patients, and no patients in this group underwent TEE.

There were a total of 25 lesions with the most common valves affected by *S. suis* infection being the aortic valve (52%) and the mitral valve (44%). Double-valve involvement (aortic and mitral) was detected in 6 patients. The median size of vegetation was 11.8 mm [IQR: 8.2–13.0]. *S. suis* IE resulted in significant valvular regurgitation (moderate or greater) in 20 (80.0%) lesions. Ten patients (52.6%) had perivalvular complications from IE with valvular perforation being the most common complication. There was one patient who had both valvular perforation and perivalvular abscess. The median hospital length of stay in the IE group was longer than in the non-IE group (28.9 days [IQR: 16–40] vs. 14.5 days [IQR: 4–22], *p* < 0.001). Thirteen of 19 patients (68.4%) required valvular surgery (in-hospital surgery in 10 patients, and elective surgery in 3 patients). There were no deaths in the IE group, and five deaths (9.6%) in the non-IE group (*p* = 0.315). All deaths occurred in-hospital with a median time to death of 2 days [IQR: 1–23]. Four of five (80%) of the patients who died expired within 5 days of admission, which highlights the severity of the condition of these patients at the time of presentation, which was most likely due to delayed hospital referral.

## Discussion

Of the overall 71 patients with *S. suis* bacteremia, the prevalence of *S. suis* IE was 26.8%, which is comparable to the rates reported from previous studies (6.4–25.6%) [1, 2, 6]. However, those studies included all patients with *S. suis* infection, unlike our study, which included only patients with *S. suis* bacteremia. Furthermore, those studies did not report the proportion of the patients that had echocardiography performed. Although the prevalence of *S. suis* IE in our study was comparable to previous studies, we think the prevalence rate of *S. suis* IE in our study may be underestimated since TTE was performed in less than half (42.3%) of patients, and even less for TEE (9.8%). In our practice, those patients who can be definitively diagnosed with IE by TTE and who have no suspected intracardiac complications will not undergo TEE. This may be due to a lack of awareness of the organism’s invasiveness, to our center’s high patient volume, and our center’s limited availability of echocardiographic resources and personnel.

Regarding the aggressiveness of *S. suis* IE, our results confirmed the ability of *S. suis* to severely damage cardiac valves and perivalvular structures. We found that 80.0% and 52.6% of *S. suis* IE patients had significant valvular regurgitation and perivalvular complications of IE, respectively. A total of 13 of 19 patients underwent valvular surgery according to standard guidelines [9]. A study of *S. suis* IE by Trirattanapa, et al. [4]. reported a similar rate of significant valvular regurgitation (81.4%) and perivalvular complications (35%) despite the fact that a higher rate of cardiac surgery was reported (81%). Similar to our findings, a study in *Staphylococcus aureus* reported a prevalence of *S. aureus* IE in *S. aureus* bacteremia of 22%, and perivalvular complications and cardiac intervention occurred in 23.0% and 64.2% of cases, respectively [10]. In the immediately aforementioned study, it should be noted that only cases of definite *S. aureus* IE (according to the modified Duke criteria) were included. In contrast, we included only 12 cases (63.2%) of definite IE as determined by ESC clinical criteria. This was due to the fact that *S. suis* is considered to be an atypical organism [9, 11]. As a result, blood draws and culture to evaluate for the continuous presence of the pathogen were far less common in patients with *S. suis* bacteremia. Nonetheless, considering the recent update of the 2023 Duke-International Society of Cardiovascular Infectious Diseases (ISCVID) criteria [11], all *Streptococcus* spp. (except *Streptococcus pneumoniae* and *Streptococcus pyogenes*) were added as typical organisms, and the requirement for timing or separate venipuncture was removed. If this 2023 updated Duke-ISCVID criteria was applied to our report, 19 patients (100%) would be classified as definite IE. However, if we included both pathological and clinical criteria, the proportion of definite IE would be 94.7% (18 of 19 patients) by ESC criteria, and 100% (19 of 19 patients) by the 2023 updated Duke-ISCVID criteria. That said – generally, not all suspected IE patients will undergo cardiac surgery, and the pathologic criteria will not be elucidated. Therefore, the ESC clinical criteria could only detect 63.2% of all IE patients. In contrast, the 2023 updated Duke-ISCVID clinical criteria [11] can detect up to 100% of patients. This information highlights the need to reclassify *S. suis* as a typical IE bacterium instead of an atypical IE bacterium. *S. suis* infection in China and Thailand accounts for up to 81% of *S. suis* infections worldwide [12]. This fact may be a major reason why this pathogen continues to be listed as an atypical IE bacterium.

A recent study of *Enterococcus faecalis* (*E. faecalis*) and *Streptococcus* spp. bacteremia [13] found a prevalence of *E. faecalis* IE and *Streptococcus* spp. IE of 16.7% and 7.3%, respectively. Even though the prevalence of these organisms was less than the prevalence of *S. suis* IE found in our study, these 2 organisms were recently added to the 2023 ESC IE guideline that recommends screening with TTE [8]. 

Concerning the predictors of *S. suis* IE in *S. suis* bacteremia, our univariate regression analysis revealed dyspnea, immunologic phenomena, new murmurs, and heart failure to be significant predictors of IE. We were unable to perform multivariate analysis to identify independent predictors due to both our study’s small sample size and to potential unfavorable interactions between and among factors. It should also be noted that, even though statistically significant, the confidence interval for each of the odds ratios was very wide. This suggests that some caution should be exercised when interpreting this data.

From a treatment perspective, patients with IE had a two-times longer hospital length of stay compared to the non-IE group. This lengthened hospital stay may lead to more nosocomial complications, physical and psychological deterioration, and potentially increased patient economic hardship. Fortunately, there were no deaths in our IE group when compared to a 6-month mortality rate of 26% in patients with *S. aureus* IE [10]. This may be due to different antibiotic susceptibilities between organisms, advances in treatment, and difference in the prevalence of prosthetic valve IE.

Despite the substantial incidence and aggressive nature of *S. suis* IE reported by the present study and other previously published studies [4, 7], there is still no specific recommendation for routine TTE surveillance for *S. suis* IE in patients with *S. suis* bacteremia. This may be due to the scarcity of *S. suis* IE studies compared to *S. aureus* IE studies, which has potentially resulted in the under-recognition of the invasiveness of this pathogen. We think the results of this study will help to reshape our perception and understanding of this pathogen, and that positive changes in clinical practice guidelines will be thusly effectuated. A good start may be to amend all IE guidelines by reclassifying *S. suis* from an atypical IE pathogen to a typical IE pathogen.

### Limitations

This study also had several limitations. First, our study’s retrospective cohort design rendered it vulnerable to confounding factors that could not be adjusted and that could adversely affect the outcomes of the study. Second, the quality of the data may be suboptimal since it was the retrospective cohort, some important data may be overlooked or not noted in patient’s files. Furthermore, we were unable to establish that some findings, such as new murmur and immunologic phenomena, which were not found in non-IE group, were systematically properly explored. Third, the sample size was small due to the low incidence of *S. suis* bacteremia even though the data was collected over an almost 16-year period, and our center is the largest national tertiary referral center in Thailand. Fourth, to find the true prevalence of *S. suis* IE in patients with *S. suis* bacteremia, all patients should have echocardiography performed. However, this was not possible at our center due to resource restrictions that include a high patient volume and a shortage of sonographers and echocardiologists. Fifth, factors that were found to significantly predict *S. suis* IE from univariate regression analysis had a very wide CI, which suggests that our data/findings be interpreted with that in mind. Sixth and last, since the prevalence of *S. suis* and its clustering distribution vary widely between and among countries, the generalizability of our findings may be limited – especially in countries with a low prevalence of *S. suis*.

## Conclusion

The results of this study revealed a 26.8% prevalence of *S. suis* IE in patients with *S. suis* bacteremia, and there were high rates of both valvular damage and perivalvular complications. We recommend that echocardiography may be indicated to evaluate for *S. suis* IE in patients diagnosed with *S. suis* bacteremia. Reclassification of *S. suis* from an atypical IE pathogen to a typical IE pathogen should be considered.

## Data Availability

The data analyzed during the current study are not publicly available due to human data privacy’s law, but are available from the corresponding author on reasonable request.

## Abbreviations

95%CI: 95% confidence interval
COA: Certificate of approval
*E. faecalis*: *Enterococcus faecalis*
ESC: European Society of Cardiology
et: Al. And others
IE: Infective endocarditis
IL: Illinois
Inc.: Incorporated
IQR: Interquartile range
ISCVID: International Society of Cardiovascular Infectious Diseases
mm: Millimeter(s)
OR: Odds ratio
*S. aureus*: *Staphylococcus aureus*
*S. suis*: *Streptococcus suis*
SD: Standard deviation
Si: Siriraj
SIRB: Siriraj Institutional Review Board
SPSS: Statistical Package for the Social Sciences
TEE: Transesophageal echocardiography
TTE: Transthoracic echocardiography
USA: United States of America
v.: Version
